# Applying the PDSA cycle to a group activity promoting lifestyle change for the active ageing of older Thai adults – a focused ethnography

**DOI:** 10.1186/s12877-022-02775-4

**Published:** 2022-02-11

**Authors:** Manothai Wongsala, Sirpa Rosendahl, Pornpun Manasatchakun, Els-Marie Anbäcken

**Affiliations:** 1 School of Health, Care and Social Welfare, Mälardalen University, Hamngatan 15, Box 325, 631 05 Eskilstuna, Sweden; 2 School of Health Sciences, University of Skövde, Högskolevägen, Box 408, S-54 128 Skövde, Sweden; 3 Boromarajonani College of Nursing Chiang Mai, 201 Don Kaew Subdistrict, Mae Rim District, Chiang Mai, 50180 Thailand

**Keywords:** Active ageing, Behaviour Lifestyle change, Lifestyle choice, Older adults, PDSA cycle, Thailand

## Abstract

**Background:**

The proportion of the older Thai population is increasing rapidly. Lifestyle may impact active ageing in later life. Interventions that empower older Thai adults to initiate and carry out lifestyle changes are needed. This study applied the Plan-Do-Study-Act (PDSA) cycle, a tool for improving lifestyle changes, with the aim of exploring interactions among older Thai adults when participating in group activities.

**Method:**

Focused ethnography was used based on participant observations, field notes and video recordings of 15 older Thai adults aged 62–78 years.

**Results:**

Older Thai adults faced difficulties at the beginning since they were unfamiliar with initiating and carrying out lifestyle changes according to the PDSA concept. This provided a learning opportunity enabling older Thai adults to reach their individual goals of lifestyle change.

**Conclusions:**

The PDSA cycle has the potential to empower older adults in group contexts to promote lifestyle changes related to active ageing.

## Background

The older population is increasing worldwide, including in Thailand. The older Thai population is expected to reach over 25 percent in 2030 [1] and Thailand has officially stated that active ageing is a part of the national agenda [2]. Active ageing as defined by the World Health Organization (WHO) policy framework is “the process of optimizing opportunities for health, participation and security in order to enhance quality of life as people age”. The concept builds on the basic pillars of health, participation, and security [3]. The health pillar is based on the prevention of disease and disabilities; the participation pillar includes work events, voluntary activities and learning opportunities according to individual needs; and the security pillar aims to ensure the protection, safety, and dignity of older people by addressing their social, financial, and physical security [4]. Older Thai adults have their own perspectives on active ageing [5]. They also have their own perspectives on each basic pillar of active ageing, related to their desired health, participation, and security [6].

As people age, they experience various physiological, psychological, and social changes [7]. Longevity is a common wish of most people, but only if it means well-being that is a subjective perception of oneself [8]. Older persons who desire to live well may be concerned about taking care of themselves more seriously than ever. An important goal therefore is to improve habits of daily life since these impact people’s ageing. Behaviours in daily life impact people’s physical and mental health and their quality of life [9]. For persons who have an illness or older adults who face changes in physical and mental health that impact their daily activities, paying attention to maintaining a healthy lifestyle and self-care behaviours could help them regain their health [10]. Moreover, behaviour and lifestyle changes could help to bring about gains in health and well-being [11]. An individual can make a lifestyle choice for better living [12]. The shaping of individual behaviour can be described and analysed with several theories, but there are still many unanswered questions about their optimal use and validity. Although each theory presents distinct elements, there are also shared elements, such as beliefs, self-efficacy, and social influences. In addition, a theory might be appropriate to a particular context, and multiple theories might be used to design an intervention to encourage an individual behavioural change [13].

This current study was inspired by the importance of lifestyle choice in daily living to enhance the quality of life. Although daily behaviour may be a relevant word to describe how older adults live their daily lives, in this study the term lifestyle is preferred instead. Lifestyle is used here to cover the way of practical acting by older adults in daily living. However, lifestyle is closely related to behaviour.

It is difficult to change behaviour or lifestyle since it is not a simple linear process. It is complex because it requires a person to change familiar and convenient habits to an unfamiliar set of new actions. It also takes a long time for new behaviours to become habitual [14]. This can be explained by the Stages of Change Model [15], which describes six stages of change: precontemplation, contemplation, preparation, action, maintenance, and relapse. Precontemplation is the stage at which individuals are neither aware nor concerned about what needs to be changed and are not considering any changes. Contemplation is the stage of ambivalence, thinking about change, and reviewing the pros and cons of change. Preparation is the stage of planning to change, taking steps to facilitate change behaviour and committing to taking action. Action is the stage of putting plans into practice and changing one’s lifestyle. Maintenance is the stage where the behaviour becomes a part of habitual daily life. The last stage is relapse, which is the stage of returning to older, unhelpful behaviour patterns [14, 15]. Imagine a person who is in the precontemplation stage and does not think about what should be changed; what could trigger that person to contemplate the new behaviours and progress to the next stages? Commonly, current behaviours are formed by a person’s long-term choices and actions until the change becomes habitual. Moreover, it is not certain that a contemplative person will prepare and act to change. It is not only difficult to motivate a person to change daily habits but necessary to think about how to maintain the new habits and avoid a relapse. Bouton [16] explained that new behaviour is difficult to sustain because it does not automatically erase old behaviour (habits), and many factors or contexts could influence people to regress to old behaviours. Therefore, this present study suggests that a person needs self-motivation to sustain the changed habits of daily life.

In addition, behaviour is influenced by capability, opportunity, and motivation, which was described by the COM-B model [17, 18]. Capability, motivation, and opportunity can influence behaviour, while behaviour can influence all three factors. Likewise, capability and opportunity can influence motivation [19]. Therefore, it is not clear which factor is the most important or should be the beginning of changing behaviours. However, it is obvious that lifestyle changes to promote active ageing which are intended to enhance quality of life are the responsibility of an individual. There is therefore a need for motivation, providing the opportunity and encouraging the capability of older adults to manage lifestyle changes on their own.

Since behavioural change is difficult and consist of several factors, an intervention that motivates supporting factors for improving behavioural change needs to be provided. An intervention could help people learn how to manage themselves. General components supporting self-management are people’s decision-making and problem-solving skills, promotion of healthy lifestyles, the use of targeted approaches, the ability to self-monitor, a definition of their own context, and the provision of opportunities to share and learn [20].

Thai health care organizations have provided various interventions for the older population to promote quality of life and maintain the value of the older population as a social asset. A strength of Thai policy is the provision of group activities, especially with older people. Self-help groups have continually aimed to improve older adults’ individual lives.

Thai society is collectivistic rather than individualistic, based on social interests rather than individual interests. Responsibility to the group promotes strong relationships while everyone takes responsibility for each other. Therefore, interventions at group level could suitably be provided for the Thai population. Another notable aspect is that Thai society perceives the role of leader as a controller rather than a colleague [21]. Older Thai adults tend to be followers when contacting health providers since they perceive the health provider as a leader. Through their roles as responsible for helping clients to improve their health and stay healthy, health providers are respected [22]. Most of the activities provided through the cooperation of older people were suggestions or information given by health personnel [1]. Believing and following health providers has a positive side, as it is easy to invite them to participate in interventions, but this could entail a risk of not matching the needs of older adults and cause them to be passive consumers. There are several studies about using programmes that tended to be of the kind that motivated individual older adults to improve their daily lifestyle, such as a study using the Physical Activity Intervention Programme [23], the Intervention Programme for Depressive Symptoms [24], and the lifestyle intervention programme enhancing long-term health-related behaviour improvement [25]. However, all the programmes mentioned in previous studies were operated by staff that acted as coaches and educators and lacked initiatives from older adults themselves. Despite good intentions, suggestions given by experts in health care may result in passivity among older adults. It was not clear that these interventions motivated thinking about change and provided opportunities for older Thai adults to gain their capacity to manage lifestyle changes.

This current study uses the Plan-Do-Study-Act (PDSA) cycle, as it proceeds from the individual’s own definition of need and goals for lifestyle changes*.* The PDSA cycle is known as the Deming cycle and originates from industry but it has also been used in healthcare improvement, according to systematic reviews by Taylor et al. [26] and Knudsen et al. [27]. It provides a structure for iterative testing of changes that improve the quality of systems for the continual improvement of a product or a process. The PDSA cycle consists of a four-step cycle. The Plan (P) step aims to identify what needs to be changed and to form a goal of change and state what to do and how to carry it out. The Do (D) step involves carrying out the plan, which includes the activities that lead to the goal. The Study (S) step provides the chance to examine the success of the change and what in the process should be adjusted and how. The Act (A) step aims to identify adaptations, modifications, and refinements to provide a new cycle of PDSA. These four steps are iteratively repeated as part of a never-ending cycle of continuous improvement [26].

The PDSA cycle is widely used to improve the quality of organizations but has been less used to improve well-being and quality of life in individuals [26, 27]. There are however examples using the PDSA cycle to encourage individual lifestyle change through group activities to improve quality of life among older adults. One such example, is the “Passion for Life” project which was successfully conducted in Sweden [12]. The PDSA cycle was applied in meetings called “Life Cafe” with groups of senior participants. Another similar project was conducted in the UK [28]. The projects encouraged older adults to manage their lifestyle change to improve their quality of life. The idea behind the concept of the PDSA cycle is that a defined goal is reachable through small steps of change, which in turn, when applied, can contribute to a sense of increased self-confidence in the individual. The iterative nature of testing the changes helps to minimize the resistance to change and supports the permanency and continuity of a new habit. In addition, the PDSA cycle focuses on enhancing the older adults’ personal capacity to plan, set a goal, carry out, and evaluate. Moreover, since it uses small steps of change and consists of just four easy steps, the PDSA cycle can easily be used by older adults themselves without moderation by health personnel.

Another advantage of the PDSA cycle is that it supports the group dynamics. All steps of the PDSA cycle encourage participants/group members to learn from each other, especially in the Study step. This provides an opportunity to discuss and encourage each group member to independently define a goal (Plan step) and change and/or continue activities (Act step).

In Thai society, with its collectivist culture [21], older adults tend to need encouragement to manage their own lifestyle choices, and most activities for the older population are provided on group level. Group activity in the style of the PDSA cycle may provide the tools needed to open up for individual improvement. Thus, an intervention using the PDSA cycle could be an appropriate lifestyle improvement concept/model to use in order to promote active ageing for older Thai adults. In Thailand, it is pioneering to use the PDSA cycle to promote older Thai adults’ quality of life, and this study therefore focuses on how the PDSA cycle works with older Thai adults as a health promotion tool in group settings. The present study emphasizes how important and necessary it is for older persons’ own voices to be heard with regard to how an intervention has the potential to encourage the capacity to manage lifestyle choices to improve quality of life. More specifically, the interactions may reveal in what way the PDSA cycle supports older Thai adults to make lifestyle changes.

## Aim

To explore interactions among older adults participating in group meetings using the PDSA cycle.

## Method

The meetings applying the PDSA cycle were conducted with a qualitative design and methodology. The interaction in this study refers to both verbal and non-verbal communication, including behaviours and actions over the period when the PDSA cycle was implemented. Since the task of this study was to describe interactions as a kind of culture among people, ethnography was used. Ethnography is a qualitative research design that is used to describe and interpret the shared and learned patterns of values, behaviours, beliefs, and languages of a culture-sharing group [29, 30]. An applied method called focused ethnography was selected for this study. While ethnography commonly studies an entire social field with a broad research purpose, where researchers who are not familiar with cultures have to gain knowledge from engagement in the field and a long-term approach is needed, focused ethnography presents a different approach. Focused ethnography engages with well-defined phenomena and issues within a specific context and is often carried out by experts who are already embedded in the field of study or are very familiar with it. It is also suitable for exploring experiences by the researcher during participant observations [31]. Focused ethnography studies episodes in social fields and has a clear specific research focus. The researcher is familiar with the culture and has background knowledge prior to data collection. Moreover, focused ethnography is also applicable in a short-term approach [32]. It is valuable for researching healthcare issues because it can link everyday interactions and wider cultural formations through its emphasis on context [33]. This study had a clear specific focus on interactions among participants. MW, who collected data, was familiar with the culture of this setting and participants, and the meetings were conducted during a relatively short time.

### Study context and participants

The PDSA cycle was applied to the meetings called “Lomwong Saangsook”, named by the first author. The name in Thai refers to meeting to build happiness together. The meetings were conducted in the conference room of a Subdistrict Health Promoting Hospital located in the north-eastern part of Thailand. This type of healthcare unit plays the most important role as the first-line health promotion and healthcare service in the Thai community. Participants included 15 older adults aged 62–78 years old with an average age of 68.3 years (Table 1). They were residents in three villages within the catchment area of the hospital and were familiar with the hospital and its staff. All participants met the inclusion criteria of being aged 60 or above, without severe illness, able to communicate without difficulties and willing to participate in the study. Around 20 older adults were qualified and informed about participation in the research project by a health provider working in the setting. Of the 20 prospective participants, 15 agreed to participate and were invited while five persons declined.

**Table 1:** Participant characteristics

Characteristic	Number
Age	
60-65	7
66-70	4
71 & above	4
Gender	
Male	8
Female	7
Married status	
Married	7
Widowed	8
Education	
Primary school	14
Secondary school	1
Occupation	
Gardener	2
Self-employed	3
Retried	7
Housekeeper	3

The Lomwong Saangsook was conducted four times at intervals of three weeks, as shown in Table 2.

**Table 2:** Stage of LS Meeting using PDSA cycle

Stage	Steps	Activities
Meeting 1	Introduction	1. Introduction to the concept of the PDSA cycle 2. Mini-lecture about healthy diet (pillars of health)
	Plan	1. Each participant decided on a plan, a goal, and a way to carry out the plan as regards a healthy diet. 2. Each participant expressed this to the group
	Do	Each participant carried out the plan for a healthy diet at home.
Meeting 2	Study	Each participant described what and how she/he had done as regards the healthy diet to check the validity of the plan for signs of progress and success or problems for improvement.
	Act	Conclusions about learning generated by the previous step, then adjusting the goal, changing methods, or continuing to practise a healthy diet.
	Plan	Each participant decided on a plan, a goal, and a way to carry out the plan as regards exercise.
	Do	Each participant carried out the plan to exercise at home.
Meeting 3	Study	Participants described what and how they had done in terms of exercise and discussed in the group how to improve activities or change goals.
	Act	Conclusions about learning generated by the previous step, then adjusting the goal, changing methods, or continuing to exercise.
	Plan	1. Mini-lecture by moderator about participation (second pillar of active ageing) and setting a plan. 2. Each participant decided on a plan, a goal, and a way to carry out the plan as regards participation. 3. Each participant expressed her/his plan to the group.
	Do	Each participant carried out the plan as regards participation at home.
Meeting 4	Study	Participants expressed what and how they had done in terms of participation and discussed in the group how to improve activities or change goals.
	Act	Conclusions about learning generated by the previous step, then adjusting the goal, changing methods, or continuing participation.
	Plan	1. Mini-lecture by moderator about security (third pillar of active ageing) and set a plan. 2. Each participant decided on a plan, a goal, and a way to carry out the plan as regards security. 3. Each participant expressed her/his plan to the group.
	Do	Each participant carried out the plan as regards security at home.
	Study	Participants expressed what and how they had done in terms of security and discussed in the group how to improve activities or change goals.
	Act step	Conclusions about learning generated by the previous step, then adjusting the goal, changing methods, or continuing practice as regards security.
	Summarize	Summarize the steps of the PDSA cycle and how participants have learnt and made lifestyle choices during participating in the meeting.

Information about the objectives of each step, and how to achieve the objectives was provided when each step started. Meetings discussing the PDSA cycle could continue to phases five, six and more, but this study was completed in four phases.

### Data collection

Data was collected through participant observations and video recordings of the meetings. Participant observations provided tools to study the process of learning new individual habits in daily life and sharing one’s learning in the group context of LS meetings. Despite the short time, this conforms well to the methodology of participant observations which is suited to study shared and learned patterns of behaviour [29, 30]. This could be matched to the PDSA cycle, which allowed members in the meetings to share and learn with and from each other in an interactive way to change habits related to lifestyle. The first author (MW) sat in the meeting room, participated and observed, and took field notes while two video cameras recorded from opposite corners. The observer (MW) who was known as a nurse instructor, responded to questions from participants during the meetings, and helped the moderator to facilitate the meetings. The digital video files were transferred to a computer. The field notes were revised in parallel with watching the videos in the evening of or one day after each meeting. The videos were used to confirm and recall what happened during the meetings and how they progressed.

### Data analysis

Video data and brief field notes (from 4 meetings which each lasted for around two hours) were entered into the computer-assisted qualitative data analysis software Atlas ti8. Codes were defined focusing on interactions among participants, and patterns of interactions were then sorted by grouping the codes. The groups of codes were defined as themes. Codes and themes were first defined by the first author and third author separately, then discussed and confirmed by all authors several times. The videos were re-watched several times by the first author, and in parts by all authors to re-observe the interactions, especially that of body language. The themes were used to create and summarize what and how the participants interacted during the meeting using the PDSA cycle. Reflective remarks memos were written during the research process to keep track of the research question.

### Ethical considerations

Participants were informed about the purpose of the study, the time required for participation, that participation was voluntary and that they could withdraw any time without giving a reason. Informed consent was obtained from participants. The participants gave their written consent to participate in the study. The names of participants were protected by referring to the participants based on gender and age. Confidentiality was maintained in all data collection and analysis processes by keeping the material in a safe place and protecting against exposure.

## Results

### Description of the empirical setting of the Lomwong Saangsook meeting

The meetings were conducted in a health care unit, which was a small two-storey building situated beside a small pond. The ground floor was divided into a few rooms providing health services, and the second floor was a meeting room. The staff of the healthcare unit who arranged the place were known to and had good relations with all participants. On reaching the place, the researchers introduced themselves to the participants and the staff of the health care unit. Female participants were sitting around a table in front of the kitchenette, while a group of men were talking in the parking area. They talked in a friendly manner to each other while waiting for others who were on the way. The gatekeeper for this study was one staff member who was well known to the first author and was helpful in recruiting the participants and assisted with the arrangement of facilities and the preparation of snacks and meals for the meetings. The research team consisted of both Thais and Swedes. The participants seemed to be interested in the two non-Thai-speaking people, who may have seemed exotic to them. In the meeting room, tables and chairs were not used. The participants sat on the small soft carpets that were laid around the room in the shape of a circle. Men and women sat separately, even though they were free to select seats. Coffee breaks were provided by us. In the later meetings, some participants brought fruit to share with each other at coffee time and gave us some fruit as a gift. The participants were given a lunch box after finishing each meeting around noon. Some had lunch together at the meeting place, while others took their lunch box back home.

The first meeting was conducted by the second author (SR) in English and was translated into Thai by the third author (PM). Authors MW and EA also participated and took field notes. The other three meetings were carried out by the Thai-speaking author alone (PM). The participants were informed about the purpose of the meetings at the beginning of each meeting, the main one being to improve active ageing through lifestyle change by the older adults themselves. The steps of the PDSA cycle were recalled several times during the meetings to inform the participants about the purpose of the current step and how they could address it. The three basic pillars of active ageing – health, participation, and security – were applied as the topics of the meetings. Although all three pillars were introduced, the participants discussed health much more than the other pillars. The observations showed that the participants found it difficult to talk concretely about the pillar participation and security. The Plan step was introduced in the first meeting and developed while participants decided what they planned to change regarding their diet/eating. The Do step was expected to be done by carrying out the plan at home. The Study and Act steps were conducted at the second meeting three weeks later. During the Study step, the participants told how the implementation of the plan was going, how they felt, and what should be changed or continued. The participants gave suggestions to each other. The moderator also suggested and motivated participants to keep carrying on and supported them if they decided to adjust their activities. In the Act step, some participants decided to adjust the way they were carrying out the plan, while some of them decided to continue the same activities. All four steps of each topic could be repeated in parallel with the next cycle of the next topics if needed. For example, while talking about how exercise had been implemented in the Study step of the third phase of the meeting, some participants could mention or discuss the healthy diet that was planned at the first meeting.

The results consist of three themes: (1) I don’t know what needs to change, (2) Learning from each other, and (3) I will show what I have done versus how I can follow him, which are exemplified below.

### I don’t know what needs to change

Since the first step of the PDSA cycle involves planning to change, the first requirement was to determine what should be changed. When introducing the idea of doing individual improvement work according to the PDSA cycle, i.e. by setting a goal and planning how to reach the goal, many participants expressed that everything was sufficient, and no change was needed:

*“I don’t know what needs to change. I always have regular meals, it is good, nothing is lacking for me” (Male, 69).*

Signs of worried expressions were observed on their faces, and they looked at each other. Instead of expressing a plan, one participant talked about the daily meal.

*“Uhhh…(silent for a while) I have eaten various types of vegetables and fish. I have eaten as others do (Male, 77).*

Almost all participants responded in the same way. The group was silent for a while. The moderator seemed worried and had a look of stress trying to clarify that the purpose of this step “Plan” was to define new activities that had never been done before. Some participants still said that they usually eat good food, and there was nothing that needed to be changed. The moderator explained that this step was to decide to do something new and different from ordinary daily living. Examples such as drinking more water, reducing coffee intake, and other small eating behaviours/habits were discussed, and it was emphasized that starting with a small thing is a good method. The participants then had a small group talk and began to look more relaxed. Once a participant made a decision and expressed an individual plan, the others also expressed their own. However, two of them could not decide, and the moderator avoided forcing them to do so.

Although the process seemed to stall at the beginning, the PDSA cycle continued with support from the moderator. In the later phases of the meeting, the participants looked more relaxed and had an easier time dealing with the meeting process. Through the group process and learning of participants, the difficulty decreased. In the later phases of the meeting, from their facial expressions and ways of talking it could be seen that it became easier for participants to understand as they became more familiar with using the PDSA cycle.

### Learning from each other

Most participants could set individual plans but lacked an exact goal or a way to measure it. For example, one participant decided to reduce the amount of coffee, but did not clarify how many cups per day to drink, while another mentioned eating a greater variety of meals without clarifying how the plan would be carried out.

*“I always drink coffee every day. I will drink less coffee” (Female, 62).*

*I think I have not eaten varied enough. I will have more varied meals (Male, 70).*

However, the Plan step could be ended here and be continued in the next meeting. In the second meeting, the Do step was discussed, and a new goal (the Plan step) was also defined. The participants and moderator were relaxed and gave each other friendly greetings. Each participant expressed what and how they had done at home and discussed finding a way to improve their plans. The Study step (in all meetings) then provided a chance to improve each individual’s own plan, such as clarifying the goal. Examples of clarifying individual goals were presented, such as counting the number of glasses or the amount of water consumed per day and measuring by using 600 ml bottles. The following excerpt shows the Do step:

*“I have drunk water, one glass after I wake up, one after breakfast, one with medicine, two or three in the daytime, two in the evening, and a glass before going to bed” (Female, 70).*

*“I prepare drinking water in a 600 ml bottle, then I know how many bottles I have drunk, it was at least one litre and a half per day” (Female, 63).*

When one participant declared his or her own goal it served as a guideline for others. Instead of mentioning only what they would do, they clarified measurable goals, such as “reduce drinking coffee from three cups to one cup per day, eating at least three kinds of fruit a week, drinking black coffee instead of the mixed three in one (sugar, cream, and coffee).

*“I commonly drink the mixed three in one coffee. I will drink black coffee instead” (Male, 69).*

This was not only an example of clarifying the goal of an individual plan but also guided two participants who made no individual plans at the first meeting to set their own plans. By learning in the Study step, it became easier to address the Act step (second, third, and fourth meeting), which involved adjusting the decision or continuing to implement the plan. Once they understood the PDSA cycle and learned that it was all right to end up without any plan at the first meeting but to develop one later, they became relaxed and more confident. For example, a participant not only said that they planned to swing their arms but also mentioned “a thousand times per day”, while another planned to do it “30 minutes per day”. Other participants did not forget to mention the details regarding “what” and “how” in their plans.

The visual observations showed that it also became easier to set plans. Verbalizing plans was suggested by the moderators to encourage the silent ones to talk and decide. Apart from the processes mentioned, the individual characteristics of the participants also seemed to matter.

### I will show what I have done versus how I can follow him

There were differences among the participants. Confident, talkative persons set and adjusted their plans more easily than silent persons did. Some participants could define their individual plans and carry them out by themselves, while some participants needed an example or suggestions. During the early phase of the meeting, many of the non-assertive participants looked worried and decided to copy others, even if their real needs were not reflected in others’ plans. Some confident participants attempted to present what they had done proudly.

When a man who always exercised showed what he did and argued that he should continue doing that, it was decided that nothing was to be changed. Most male participants said they wanted to exercise like him. It may be safer to have something in hand.

*“I will do body stretches like this (showed how he exercises). I think it is good to continue with it” (Male, 72).*

*I will do exercises with body stretches the same as Mr. S. (Male, 65).*

The situation of plans without clarified goals and how to measure them appeared again.

However, all participants had improved in their own way; for example, after discussion, the man above decided to perform shadow boxing instead of following others.

## Discussion

The aim of this study was to explore the interactions among older adults when they participated in meetings using the PDSA cycle to motivate lifestyle change. The findings are discussed in relation to the concepts described in the background drawing on previous research. The findings illustrated interactions showing that the PDSA cycle has the potential to promote lifestyle change managed by older Thai adults themselves. Although the participants were not familiar with initiating activities by themselves, which caused difficulty at the beginning, they could learn and adapt more easily in subsequent sessions. One way of making this process easier was beginning from a small topic that was easy to carry out. Although the PDSA cycle could allow participants to select any topics independently, by focusing on the pillars of active ageing, the meetings offered the same topics for all participants. Nonetheless, they could decide their plans and goals and how to carry them out by themselves.

While asking these older adults to set individual plans for lifestyle change, the moderator sometimes faced difficulty when participants said that nothing needed to be changed. Contemplating the motivation that improving life is an important goal is a promising start. The meetings provided a mini-lecture to inform and motivate participants to think about their well-being. The Plan step of the PDSA cycle also encourages older adults to contemplate and prepare for change and then express it to the group. Listening to other participants could influence contemplation of some changes, even if their plans were already good enough, the plans could also be changed for the better. While the Do step provided a chance to maintain the action, the Study and Act steps provided an opportunity to adjust individual plans together with the other study participants. This approach provides a social context that supports maintaining new behaviours and resists relapse. Each participant will be a witness and learn from each other, which may prevent relapsing to old behaviours. In addition, older Thai adults also have their unique perspective on health, participations, and security [6]. However, the health pillar seems to be the most important to discuss for these older Thai adults. They seemed to find the pillars participation and security too abstract. The PDSA cycle might support them to make a choice of improved lifestyle in their own way. In addition, since Thai older adults tend to be followers when in contact with health providers [22], the PDSA cycle has the potential to encourage older Thai adults to become active in lifestyle choices to enhance their quality of life.

The PDSA cycle promotes the capacity, provides an opportunity, and gives motivation in much the same way as the COM-B model [17, 18]. It promotes the capacity of older adults to manage their own life and provides opportunities for self-improvement among groups of people as a supporting social context. It also motivates them to think about having an improved life and being confident, which leads to the beginning of lifestyle changes for quality of life.

The PDSA cycle can be used individually, but it is well suited for use in groups according to Gilbertson and Batty [28] and Nilsson [12]. In addition, the PDSA cycle has its own advantage as a learning tool since its’ steps provide opportunities for learning, especially at a group level. The findings showed that the participants functioned well in the group process. They learned from examples, made promises and were witnesses for each other. This is in accordance with a collectivistic way of interacting in Thai society, which considers the group more important than the individuals [21]. In addition, respecting and trusting the health provider [22] could be a barrier to being independent, but this could also make it convenient to get an activity started. However, the individual differences between the participants revealed that they had their own ways of improving. Individual characteristics should be considered when using the PDSA cycle, as this study believes that each participant has her/his own way to success. Furthermore, encouraging some participants to be moderators in the future could be considered since the observations showed that some participants acted as informal leaders during meetings. The PDSA program both in the UK [28] and Sweden [12] conducted the PDSA steps within group contexts and the content of the group meetings were similar to this study. The groups in both Sweden and the UK encouraged social interaction between the seniors and to some extent the seniors also made lifestyle changes, as seen in this study.

The PDSA cycle is suitable for practical use to promote quality of life for older Thai adults since it is available at the group level which is commonly provided in Thailand. It could be applied at a micro-level in a healthcare unit and expanded to a higher level to schools for older people and senior clubs, which are operated in all parts of Thailand.

Limitations: While the PDSA steps can be conducted many times continuously, the scope of this study allowed only four meetings. Despite this limitation, the aim of the study could be achieved using focused ethnography. Since times to observe the interactions of participants were limited, the field notes are rather brief. The video recordings were provided to keep more details and making it possible to re-observe. Language difference among authors could be noted, and video recordings were conducive to confirm the meanings between Thai and non-Thai-speaking researchers. Another limitation was that the participants did not choose the topics by themselves but were instead encouraged to make changes related to the pillars of active ageing [3]: health, participation, and security. Because the target group for the testing capability of the PDSA cycle to be applied as a tool to promote lifestyle changes is older Thai adults, active ageing, which is emphasized by national Thai policies, prompted the choice of these topics. Apart from encouraging these older adults to set their individual plans freely related to the topics given, the subjective views of the three basic pillars among older Thai adults were elucidated [6].

## Conclusion

The PDSA cycle has the potential to be applied as a tool for lifestyle change. It is available for group activity according to its own process and can be a pioneer for motivating self-management among older Thai adults. Meetings applying the PDSA cycle can be considered by policy makers as a tool to promote active ageing in the healthcare system in Thailand. Health personnel who are expected to apply this tool need to be trained since some challenges and opportunities to improve lifestyle change were shown as examples. Moreover, activity providers need to be concerned about older Thai adults having their own perspectives [6] and the potential to take responsibility themselves to improve their quality of life by making their own choices for lifestyle change.

Suggestions for further study: This study found that some participants had the capability that could make them suitable as motivators and moderators conducting PDSA cycles. A further study could evaluate the possibility of holding meetings using the PDSA cycle that are conducted by older adults themselves. Participants own choice of themes for minilectures and choices for lifestyle changes also need to be studied further, which interactions among participants revealed in this study.

## Data Availability

The datasets generated and/or analysed during the current study are not publicly available due to the restrictions of our ethical vetting, and as this is an ongoing doctoral research project with unpublished data, they are available from the corresponding author on reasonable request.

## Abbreviations

WHO: World Health Organization
COM-B: The behaviour change wheel consists of capability, opportunity, motivation, and behaviour
LS Meeting: Lomwong Saansook meeting
PDSA: The Deming Cycle which consists of four steps: Plan-Do-Study-Act
